# An Area-Efficient up/down Double-Sampling Circuit for a LOFIC CMOS Image Sensor

**DOI:** 10.3390/s23094478

**Published:** 2023-05-04

**Authors:** Ai Otani, Hiroaki Ogawa, Ken Miyauchi, Sangman Han, Hideki Owada, Isao Takayanagi, Shunsuke Okura

**Affiliations:** 1 Research Organization of Science and Engineering, Ritsumeikan University, 1-1-1 Noji-Higashi, Kusatsu 525-8577, Japan; ri0096xe@ed.ritsumei.ac.jp (A.O.);; 2 Brillnics Japan Inc., 6-21-12 Minami-Oi, Shinagawa-ku, Tokyo 140-0013, Japan

**Keywords:** CMOS image sensor, LOFIC, HDR, read-out chain, small area, double-sampling

## Abstract

A lateral overflow integration capacitor (LOFIC) complementary metal oxide semiconductor (CMOS) image sensor can realize high-dynamic-range (HDR) imaging with combination of a low-conversion-gain (LCG) signal for large maximum signal electrons and a high-conversion-gain (HCG) signal for electron-referred noise floor. However, LOFIC-CMOS image sensor requires a two-channel read-out chain for LCG and HCG signals whose polarities are inverted. In order to provide an area-efficient LOFIC-CMOS image sensor, a one-channel read-out chain that can process both HCG and LCG signals is presented in this paper. An up/down double-sampling circuit composed of an inverting amplifier for HCG signals and a non-inverting attenuator for LCG signals can reduce the area of the read-out chain by half compared to the conventional two-channel read-out chain. A test chip is fabricated in a 0.18 μm CMOS process with a metal–insulator–metal (MIM) capacitor, achieving a readout noise of 130 μ$V_{\mathrm{rms}}$ for the HCG signal and 1.19 V for the LCG input window. The performance is equivalent to 103 dB of the dynamic range with our previous LOFIC pixel in which HCG and LCG conversion gains are, respectively, 160 $\mu {V/e}^{-}$ and 10 $\mu {V/e}^{-}$.

## 1. Introduction

In the development of Internet of Things (IoT), complementary metal oxide semiconductor (CMOS) image sensors are expected to be used under extreme-illumination conditions, e.g., outdoors, in which a high-dynamic-range (HDR) CMOS image sensor is required to prevent objects from becoming overexposed or underexposed. For instance, the intra-scene dynamic range required to cover both an LED sign and a person on a sidewalk in a night scene at a local town is over 86 dB [1]. The intra-scene dynamic range is defined as the ratio between electron-referred noise floor and maximum signal electrons within a single image [2]. Furthermore, an intra-scene dynamic range of greater than 100 dB is required for driver-assistance systems in automotive vehicles [1,3,4].

**Figure 1:**
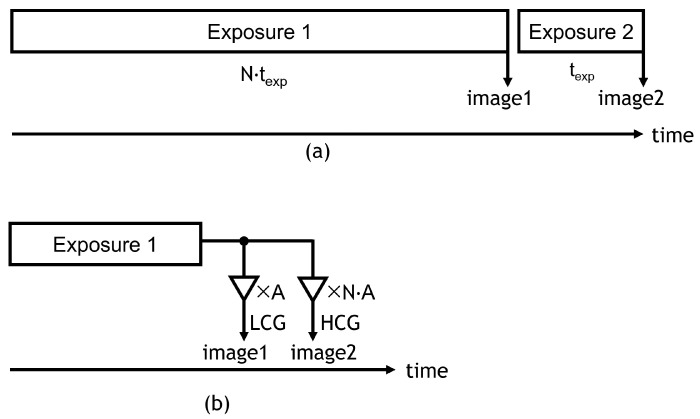
Image generation methods of MEHDR and SEHDR. (**a**) MEHDR; (**b**) SEHDR.

**Figure 2:**
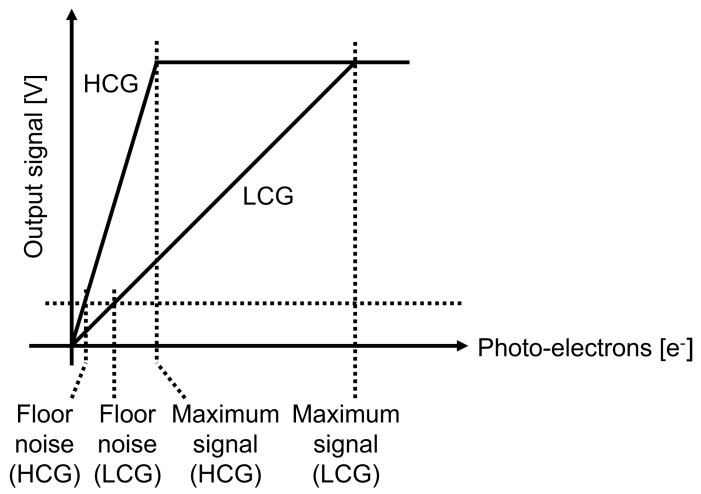
Photo−conversion characteristics of LOFIC−CMOS image sensors.

In order to realize HDR CMOS image sensors, many approaches have been proposed. Non-linear response approaches such as logarithmic compression [5,6,7] and knee compression [8] can reduce the data bandwidth because of their low bit-resolution, but are not preferred in terms of complex image signal processing. Linear response approaches such as multiple-exposure, high-dynamic-range (MEHDR) [9,10,11,12] and single-exposure, high-dynamic-range (SEHDR) approaches are preferred for simple image-signal processing. Even though the MEHDR approach is widely adopted, MEHDR images that combine two images taken at different exposure timings will be distorted due to the misalignment of the timings, as shown in Figure 1a. Thus, the SEHDR approach is becoming increasingly attractive. SEHDR is realized with dual conversion gain (DCG) pixels [13,14,15] or lateral overflow integration capacitor (LOFIC) pixels [14,15,16,17,18,19,20,21,22,23], in which the low-conversion-gain (LCG) signal and high-conversion-gain (HCG) signal are combined, as shown in Figure 1b. In DCG pixels, the photoelectrons integrated in a photodiode (PD) are read out twice in different gains, namely LCG and HCG. Since the electron-referred noise is low for the HCG, even though the maximum number of the electron readout is limited due to a given signal voltage range, as shown in Figure 2, the pixel signal under low illuminance is visible. On the other hand, since the maximum number of electrons in the read-out at LCG is high, as shown in Figure 2, the pixel signal under high illuminance is extended. Thus, the combined signal can achieve HDR with low read-out noise and large PD full-well capacity. The LOFIC pixel utilizes a similar two-times read-out scheme, HCG and LCG, and can achieve a higher dynamic range than DCG pixels because the photo-electrons are integrated not only in the PD but also in high-density capacitors. However, the circuit area for the LOFIC pixels is large due to the two-channel read-out chain for polarity-inverted LCG and HCG signals [20,22,23], while the DCG pixel signal has a one-channel read-out chain because the signal polarities of the LCG and HCG signals are the same [13]. Challenges to the read-out chain for LOFIC-CMOS image sensors are summarized as follows:

- The LOFIC pixels outputs polarity-inverted HCG and LCG signals.
- The circuit area of the two-channel read-out chain for the LOFIC pixels is larger than that for DCG pixel.
- The chip cost of LOFIC-CMOS image sensors will be high because of the large circuit area.

**Figure 3:**
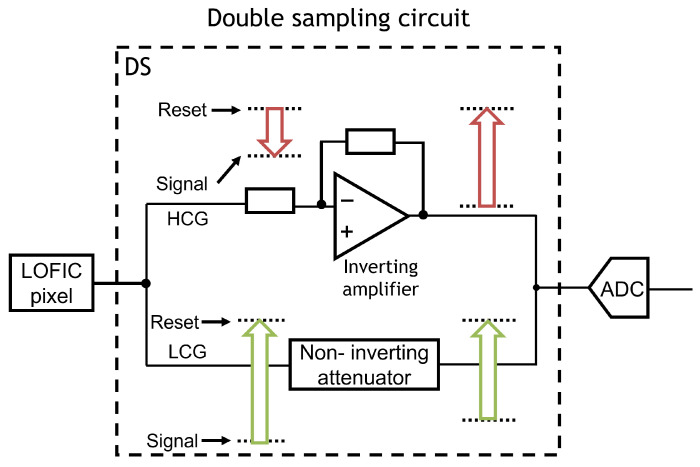
Signal processing function of the double-sampling circuit.

The read-out chain performs double-sampling for both HCG and LCG signals to cancel the pixel offset voltage and/or pixel reset noise, followed by analog/digital conversion. To realize a single-channel read-out chain and reduce the chip cost of LOFIC-CMOS image sensors, an up/down double-sampling circuit composed of an inverting amplifier and non-inverting attenuator is proposed in this paper. Figure 3 represents the signal processing function of the double-sampling circuit.

The pixel HCG signal, which is the voltage difference between the reset level and signal level from LOFIC pixel, is read out with the inverting amplifier for low-input referred circuit noise. The pixel LCG signal, which is the voltage difference between reset level and signal level from the LOFIC pixel, is then read out with the attenuator to increase the maximum signal range. Even though the polarity of the LCG and HCG signal output from the LOFIC pixel is inverted, the polarity of the input signal to the following analog/digital converter (ADC) is the same and a single ADC can process both the HCG and LCG signals. Moreover, the attenuator is merged with the ADC, such as column parallel successive-approximation-register (SAR) ADC, to further decrease the area of the double-sampling circuit. A test chip of the proposed double sampling circuit was fabricated with a standard CMOS process with metal–insulator–metal (MIM), and evaluated to estimate the dynamic range of LOFIC-CMOS image sensors with the proposed double-sampling circuit. Our contribution is summarized as follows:

- We propose a single-channel read-out chain for the LOFIC pixel to realize a small-area peripheral circuit for LOFIC-CMOS image sensors.
- We present a design methodology of signal voltage range from the pixel to the ADC to maximize the effective full-well capacity (FWC) of the LOFIC-CMOS image sensors.
- We estimate the LOFIC pixel dynamic range with the proposed up/down double-sampling circuit based on the measurement results of a fabricated test chip.

Section 2 provides a basic pixel schematic and the operation timing of the LOFIC pixel. The proposed single-channel read-out chain with the up/down double-sampling circuit is described in Section 3, followed by the design and measurement results of a fabricated test chip shown in Section 4. Section 5 provides a summary of this paper.

## 2. Lateral Overflow Integration Capacitor (LOFIC) Pixel

**Figure 4:**
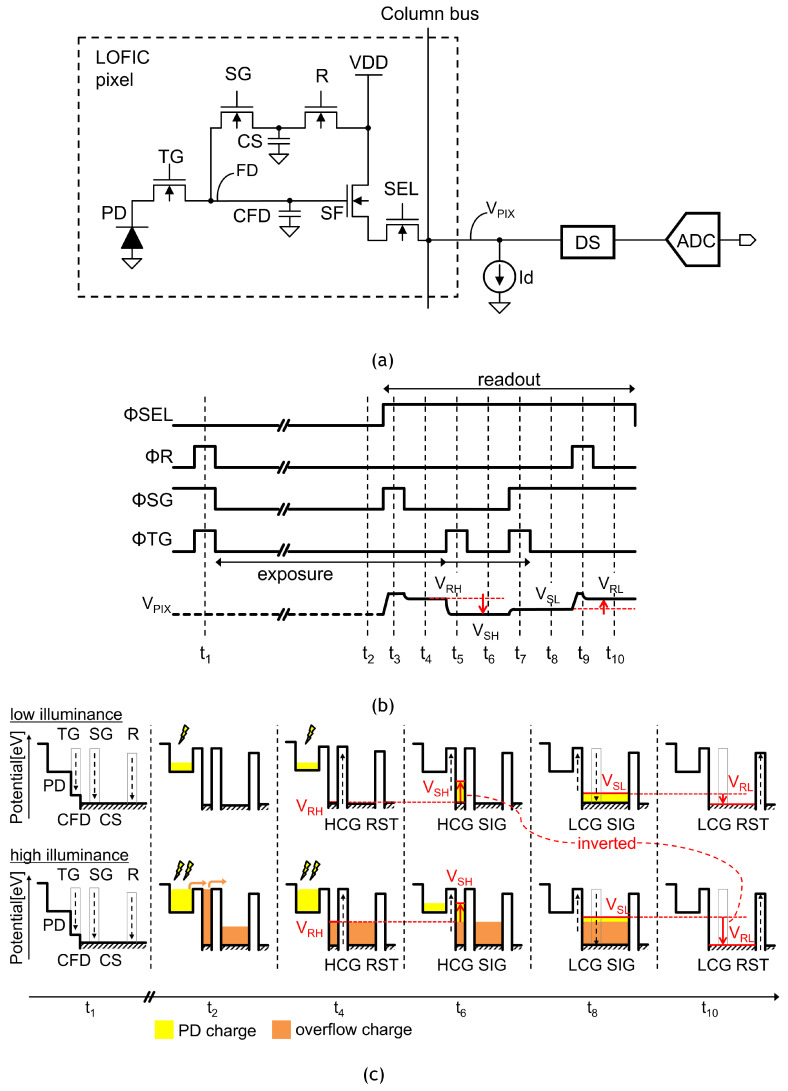
Circuit schematic, pixel operation timing and potential diagram of a lateral overflow integration capacitor (LOFIC) COMS image sensors. (**a**) Pixel circuit schematic of a LOFIC-CMOS image sensor; (**b**) pixel operation timing of LOFIC-CMOS image sensors; (**c**) potential diagram of LOFIC-CMOS image sensors.

Figure 4a shows a schematic diagram of the LOFIC pixel that is composed of a photodiode PD, a transfer gate TG, a switching gate SG, a reset gate R, a source-follower transistor SF, and a select transistor SEL, the parasitic capacitance on FD node CFD, and a charge storage capacitor CS. The double-sampling circuit, DS, derives the voltage difference between a reset level ($V_{R}$) and a signal level ($V_{S}$) from LOFIC pixel, and the voltage difference is then digitized by an ADC. The photo-electrons from the pixel are read out twice by SF, which is composed of a source-follower transistor and bias (Id). First, the photo-electrons integrated in the PD are read out in the HCG mode while SG is turned off. The HCG signal is suitable for the case with low illuminance. Secondly, the photo-electrons integrated in the PD, CFD and CS are read out in the LCG mode while SG is turned on. The photo-electrons generated in PD overflow into the FD node beyond the potential barrier of the TG when the PD is saturated. Then, photo-electrons overflow into the CS capacitor beyond the potential barrier of the SG when FD is saturated. The LCG signal is suitable for the case with high illuminance.

The operation timing and electric potential diagrams of the LOFIC pixel are shown in Figure 4b,c, respectively. At the beginning of the exposure period ($t_{1}$), the transistors R, SG, and TG are turned on to reset the PD, the CFD, and the CS. During the exposure period ($t_{2}$), as shown in Figure 4c, photo-electrons generated under low illuminance are integrated in the PD, while photo-electrons under high illuminance are integrated in the PD, CFD, and/or CS. At the end of the exposure period, the pixel signal readout is provided when the SEL transistor is turned on.

The readout for the HCG reset level $V_{RH}$ is provided at $t_{4}$ after the SG is pulsed to share a dark current charge integrated in the small CFD with a large CS ($t_{3}$). Then, the HCG signal level $V_{SH}$ readout is provided at $t_{6}$ after the charge transfer pulse is applied to the TG gate at $t_{5}$. The voltage difference between $V_{RH}$ and $V_{SH}$ is derived using the following double-sampling circuit. Since the same FD reset noise is included in both $V_{RH}$ and $V_{SH}$, a low-noise signal is readout under low illuminance. The conversion gain obtained by $q_{0}/CFD$ is as high as 160 $\mu {V/e}^{-}$ in our previous work [16], where $q_{0}$ and CFD are the elementary charge and parasitic capacitance for the FD node, respectively. The HCG signal shows a linear photoconversion response for low-input illuminance, in which the generated photo-electron charge is lower than the charge storage capacity of the PD. It is noted that the voltage difference between $V_{RH}$ and $V_{SH}$ is negative because the electric potential at the FD node increases during the transition from $V_{RH}$ to $V_{SH}$.

The LCG signal level $V_{SL}$ readout is provided at $t_{8}$ after the SG gate is turned on and the TG is pulsed again at $t_{7}$ to merge the photo-electrons integrated in the PD, CFD, and the CS. Then, the LCG reset level $V_{RL}$ readout is provided at $t_{10}$ after R is pulsed again to reset the CFD and CS at $t_{9}$. The voltage difference between $V_{SL}$ and $V_{RL}$ is derived from the following double-sampling circuit. Since the conversion gain provided by $q_{0}/\left(CFD+CS\right)$ is as low as 10 $\mu {V/e}^{-}$ [16], the LCG signal shows a linear photoconversion response up to a high level of illuminance. Even though the reset noise in the LCG signal is large due to the uncorrelated double-sampling in $V_{SL}$ and $V_{RL}$, the noise is not visible as long as it is lower than the optical shot noise. It is noted that the voltage difference between $V_{SL}$ and $V_{RL}$ is positive because the electric potential at the FD node reduces during the transition from $V_{SL}$ to $V_{RL}$.

A post-processing circuit selects the HCG signal if the LCG signal is lower than a given threshold voltage; conversely, it selects the LCG signal if the LCG signal is higher than the threshold voltage. The LCG signal is also multiplied for signal linearization from low illuminance to high illuminance, and an HDR image is realized thanks to the low-noise HCG signal and the high-FWC LCG signal. However, both HCG and LCG signal readouts should be provided for the post-processing stage. Thus, a two-channel read-out chain is typically utilized for HCG and LCG signals for which the polarity is inverted.

## 3. Proposed Readout Circuit

A read-out chain for the LOFIC pixel to process both HCG and LCG pixel signals is presented in this section. First, a conceptual view of the voltage domain level diagram is shown to visualize the critical voltage signal. Then, an up/down double-sampling circuit is proposed in accordance with the voltage diagram, along with the evaluation results of the test chip.

### 3.1. Voltage Domain-Level Diagram

**Figure 5:**
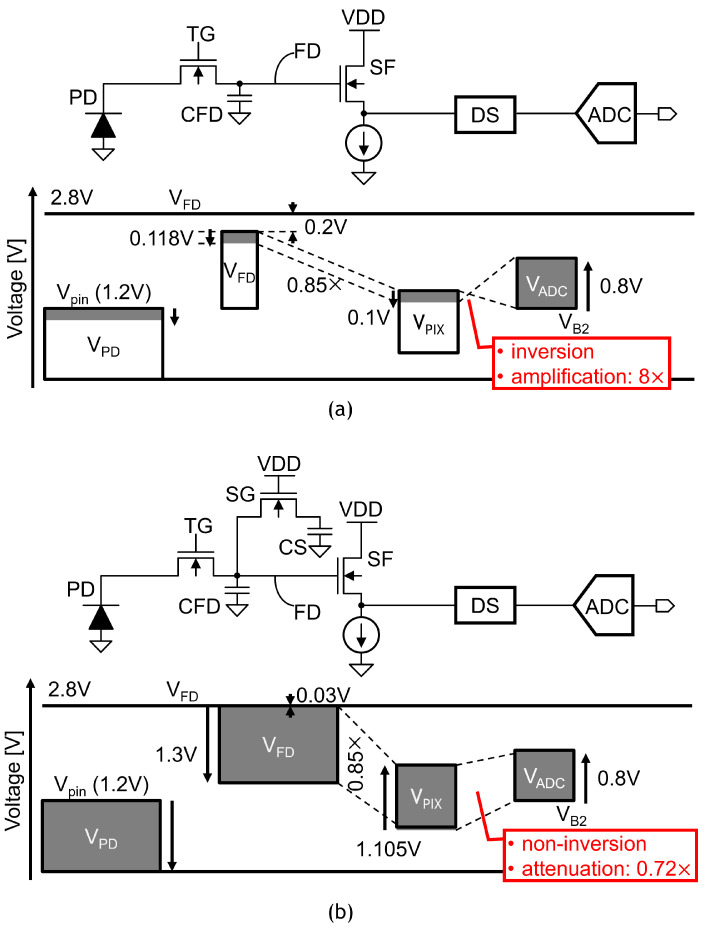
Voltage level diagram of LOFIC-CMOS image sensors used to reduce input-referred noise and to increase pixel FWC. (**a**) HCG signal: gain of double sampling circuit should be high to decrease the input referred noise at FD node; (**b**) LCG signal: gain in double-sampling circuit should be low to increase the signal swing at FD node.

For LOFIC-CMOS image sensors, a voltage domain-level diagram is designed, as shown in Figure 5. The voltage diagram is given for the pinning voltage of the PD ($V_{pin}$), the signal voltage swing at the FD node and at the pixel SF output, and the input window of an ADC.

For the HCG signal shown in Figure 5a, the voltage gain of the double-sampling circuit needs to be high enough and 8× is selected to decrease the input-referred circuit noise to achieve a high dynamic range. The input window of the double sampling circuit is therefore provided by 0.1 V for the 0.8 V ADC input window [2], and the signal voltage swing at the FD node is limited to below 0.118 V with a 0.85× source-follower gain. Even though the clock feedthrough caused on small CFDs is as large as 0.2 V when the SG is turned off, the voltage margin used to transfer the photo-electrons integrated in the PD to the FD node is large enough.

For the LCG signal shown in Figure 5b, to transfer all PD charges to the FD node, the voltage swing at the FD node is limited to below 1.3V for 1.2V$V_{pin}$, and a 2.8 V power supply voltage and voltage margin in PVT variations. It is noted that the clock feedthrough caused for CFD and CS is as small as 0.03 V when the RST is turned off. The maximum voltage swing at the FD node is set to be 1.3 V because the FWC is given by $\left(CFD+CS\right)\cdot \max\left(V_{FD}\right)$. The input window of the double-sampling circuit is given by 1.105 V with a 0.85× source-follower gain, and the gain of the double-sampling circuit is set as 0.72× for a 0.8 V ADC input range.

### 3.2. Read-Out Chain with an up/down Double-Sampling Circuit

**Figure 6:**
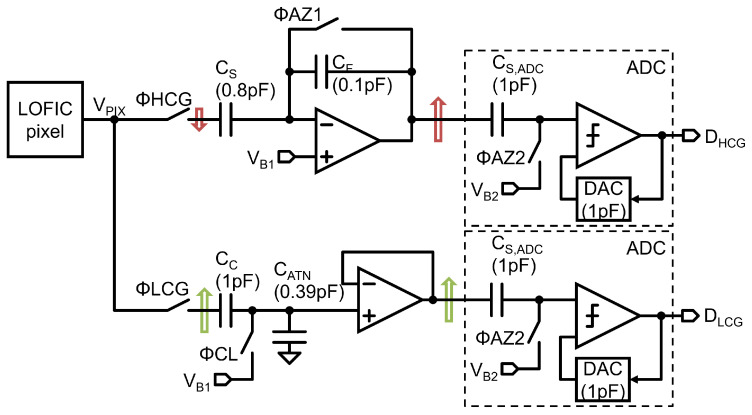
A baseline two-channel read-out chain for LOFIC pixels.

A baseline read-out chain that satisfies the voltage domain level diagram is shown in Figure 6. The LOFIC pixel output signal $V_{PIX}$ is fed to an inverting amplifier when ΦHCG is high. The voltage difference between the reset level $V_{RH}$ and signal level $V_{SH}$ is inverted, amplified by 8 times, and then digitized with a SAR ADC. The inverting amplifier is composed of a sampling capacitor $C_{S}$, a feedback capacitor $C_{F}$, and an amplifier [24,25]. The ADC is composed of a sampling capacitor $C_{S,ADC}$, an SAR capacitor digital/analog converter (DAC), and a comparator. The LOFIC pixel output signal is fed to the attenuator when ΦLCG is high. The voltage difference between the signal level $V_{SL}$ and reset level $V_{RL}$ is attenuated 0.72 times and then digitized with the ADC. The attenuator is composed of a coupling capacitor $C_{C}$, an attenuation capacitor $C_{ATN}$, and a voltage-follower [26]. Since the signal polarities of the HCG and LCG signal at the ADC input are the same, the same ADC circuit is used for HCG and LCG signals. However, this two-channel circuit requires a large area and will increase the cost of LOFIC-CMOS image sensor chips. The total capacitance of this baseline read-out chain is 6.29 pF, which can be used to roughly estimate the circuit area that is dominated by the layout area of the capacitors.

**Figure 7:**
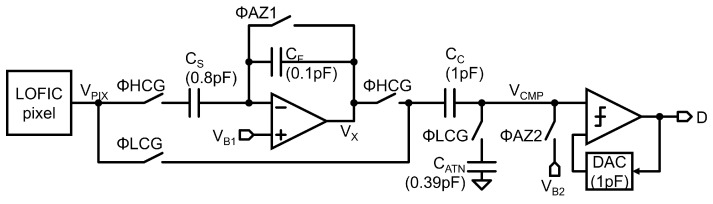
Schematic diagram of the proposed double sampling circuit.

In order to reduce the area of the read-out chain, an area-efficient read-out chain with an up/down double-sampling circuit is proposed, as shown in Figure 7. While the inverting amplifier is the same as the baseline circuit, the attenuator is merged in the ADC. The pixel output signal $V_{PIX}$ is sampled in a $C_{C}$ and attenuated with $C_{ATN}$ when ΦLCG is high, in which the coupling capacitor $C_{C}$ also operates as a sampling capacitor of the ADC. The ADC is also shared for the HCG and LCG signals. The attenuation capacitor $C_{ATN}$ is disconnected during the HCG mode when ΦLCG is turned off. The total capacitance of the proposed double-sampling circuit is 3.29 pF. The area of the proposed read-out chain will be reduced by around 48% compared to the baseline read-out chain.

**Figure 8:**
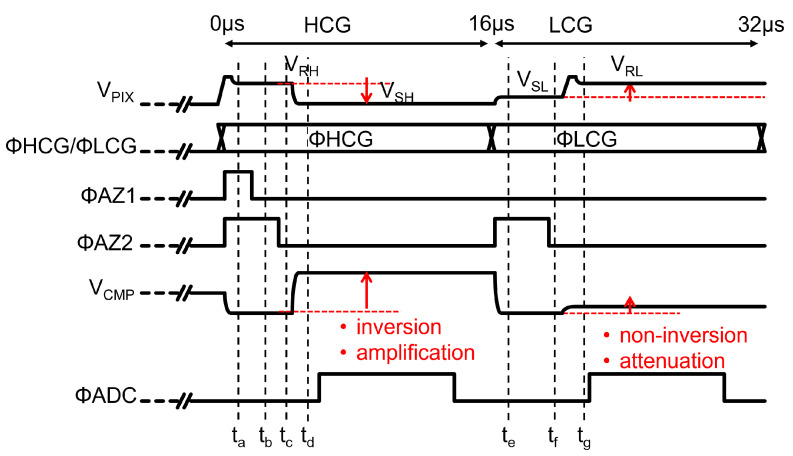
Timing diagram of the double-sampling circuit.

Figure 8 shows a timing diagram of the read-out chain with the up/down double-sampling circuit. In the HCG mode, ΦHCG is high. First, ΦAZ1 and ΦAZ2 are increased to sample the pixel reset level $V_{RH}$ and reset the amplifier and the comparator ($t_{a}$). Then, ΦAZ1 is decreased to hold $V_{RH}$ in $C_{S}$ ($t_{b}$), and ΦAZ2 is decreased to hold the bias voltage difference given by $V_{B1}-V_{B2}$ in a clamp capacitor $C_{C}$ ($t_{c}$). After the pixel output voltage $V_{PIX}$ drops to $V_{SH}$, the amplifier output voltage $V_{X}$ at $t_{d}$ is obtained as follows

(1) $$V_{X}\left(t_{d}\right)=\frac{C_{S}}{C_{F}}\left(V_{RH}-V_{SH}\right)+V_{B1}.$$

The comparator input voltage $V_{CMP}$ at $t_{d}$ is given by

(2) $$V_{CMP}\left(t_{d}\right)=V_{X}\left(t_{d}\right)-\left(V_{B1}-V_{B2}\right)=\frac{C_{S}}{C_{F}}\left(V_{RH}-V_{SH}\right)+V_{B2}.$$

After this operation, the correlated double-sampling signal $\left(V_{RH}-V_{SH}\right)$ with gain $C_{S}/C_{F}$ is converted into a digital signal with the SAR-ADC when ΦADC is high.

In the LCG mode, ΦLCG is high. First, ΦAZ2 is increased to sample the pixel signal level $V_{SL}$ and to reset the comparator ($t_{e}$). ΦAZ2 is then reduced to hold $V_{SL}$ in $C_{C}$ ($t_{f}$). After the pixel output voltage $V_{PIX}$ rises to the pixel reset level $V_{RL}$, the comparator input voltage $V_{CMP}$ at $t_{g}$ is given by

(3) $$V_{CMP}\left(t_{g}\right)=\frac{C_{C}}{C_{C}+C_{ATN}}\left(V_{RL}-V_{SL}\right)+V_{B2}.$$

After this operation, the differential double-sampling signal $\left(V_{RL}-V_{SL}\right)$ with a gain of $C_{C}/\left(C_{C}+C_{ATN}\right)$ is converted into a digital signal with the ADC. Equations (2) and (3) indicate that the signal polarities of HCG and LCG signals at the comparator input are alined.

### 3.3. Noise Analysis

In the HCG mode, the input-referred circuit noise needs to be low to achieve a high dynamic range. On the other hand, in the LCG mode, the noise requirement can be relaxed as long as the noise of the read-out chain is lower than the photon-shot noise at the switching point between the HCG mode and the LCG mode.

**Figure 9:**
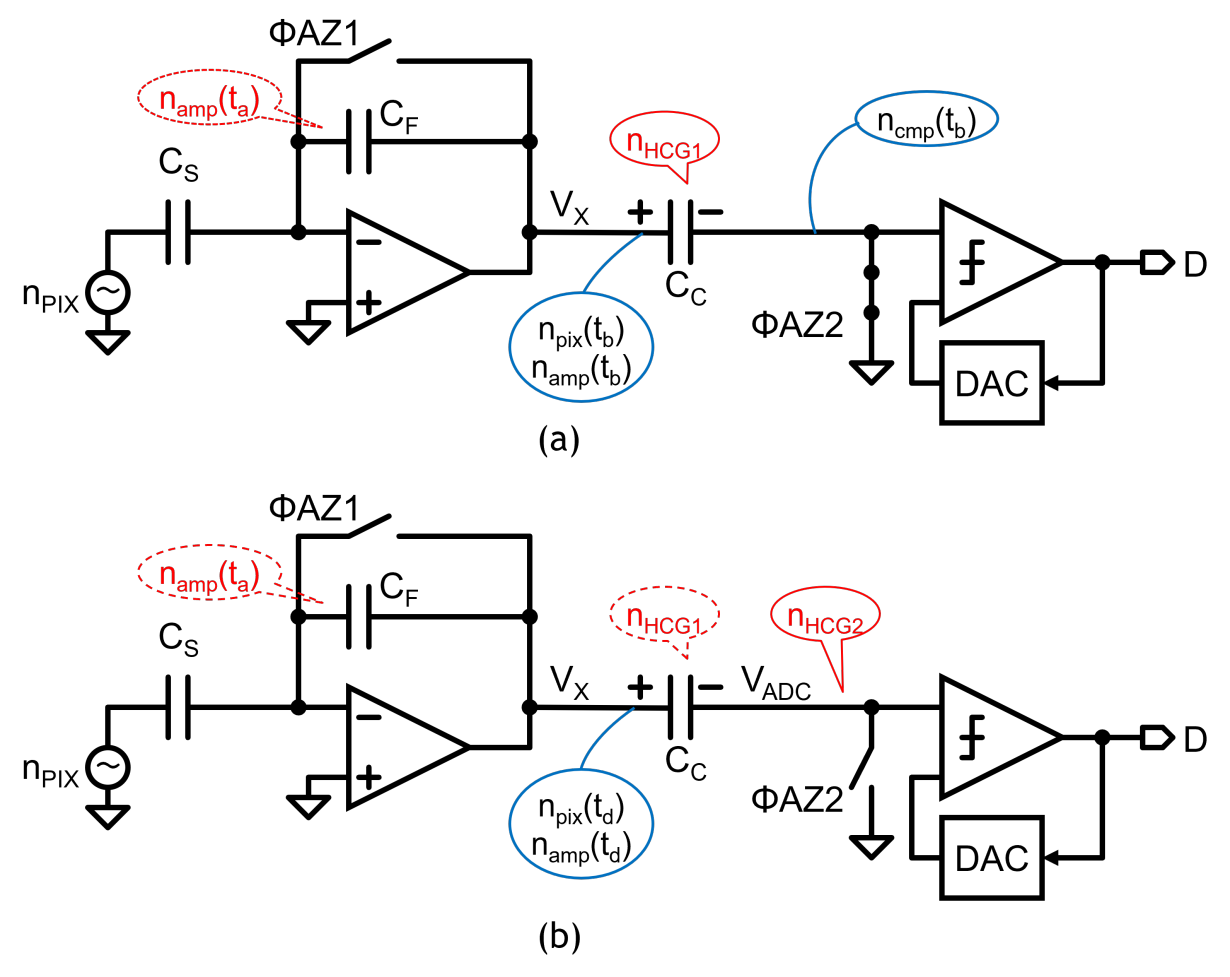
Equivalent circuits to analyze HCG noise. (**a**) Circuit and noise at $t_{b}$; (**b**) Circuit and noise at $t_{d}$.

The HCG noise is analyzed with the equivalent circuits shown in Figure 9, where $V_{RH}=0=V_{SH}$, $V_{B1}=0$, $V_{B2}=0$ for simplicity. At the sampling of $V_{RH}$ in $C_{S}$ when ΦAZ1 is turned off ($t_{b}$), the reset noise of the amplifier, $n_{amp}$, is also sampled in $C_{F}$. When ΦAZ2 is turned off, the reset noise $n_{amp}\left(t_{a}\right)$, pixel output noise $n_{pix}$, amplifier output noise $n_{amp}$ and comparator reset noise $n_{cmp}$ are sampled in $C_{C}$ ($t_{b}$). The total noise $n_{HCG1}$ in $C_{C}$, $n_{HCG1}$, is thus given by

(4) $$n_{HCG1}^{2}={\left(\frac{C_{S}}{C_{F}}n_{pix}\left(t_{b}\right)\right)}^{2}+n_{amp}^{2}\left(t_{a}\right)+n_{amp}^{2}\left(t_{b}\right)+n_{cmp}^{2}\left(t_{b}\right).$$

During the analog/digital conversion of the HCG signal, the total noise at the comparator input, $n_{HCG2}$, is given by

(5) $$\begin{array}{cc} n_{HCG2}^{2} & ={\left(\frac{C_{S}}{C_{F}}n_{pix}\left(t_{d}\right)\right)}^{2}+n_{amp}^{2}\left(t_{a}\right)+n_{amp}^{2}\left(t_{d}\right)-n_{HCG1}^{2}\\ \; & ={\left(\frac{C_{S}}{C_{F}}\right)}^{2}\left(n_{pix}^{2}\left(t_{b}\right)+n_{pix}^{2}\left(t_{d}\right)\right)+\left(n_{amp}^{2}\left(t_{b}\right)+n_{amp}^{2}\left(t_{d}\right)+n_{cmp}^{2}\left(t_{b}\right)\right). \end{array}$$

Since the amplifier reset noise $n_{amp}\left(t_{a}\right)$ is removed, the total HCG noise is simulated with the sum of the noise at $t_{b}$ and the noise at $t_{d}$. The input-referred noise is given by $\frac{C_{F}}{C_{S}}n_{HCG2}$.

**Figure 10:**
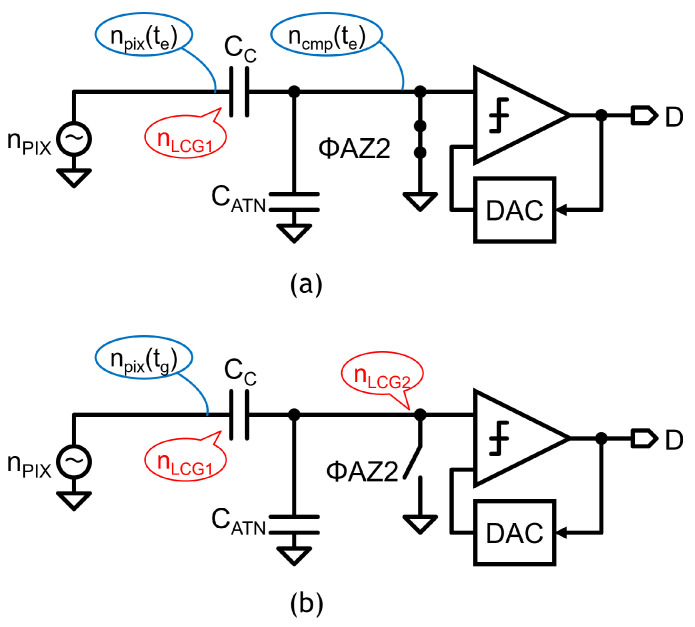
Equivalent circuits to analyze LCG noise. (**a**) circuit and noise at $t_{e}$; (**b**) circuit and noise at $t_{g}$.

The LCG noise is analyzed with equivalent circuits shown in Figure 10, where $V_{RL}=0=V_{SL}$ for simplicity. When the LCG signal voltage $V_{SL}$ is sampled in $C_{C}$, with ΦAZ2 turned off, the pixel output noise $n_{pix}$ and the comparator reset noise $n_{cmp}$ are also sampled in $C_{C}$ ($t_{e}$). The total noise $n_{LCG1}$ held in $C_{C}$, $n_{LCG1}$, is thus given by

(6) $$n_{LCG1}^{2}={\left(\frac{C_{C}}{C_{C}+C_{ATN}}\right)}^{2}n_{pix}^{2}\left(t_{e}\right)+n_{cmp}^{2}\left(t_{e}\right).$$

During the analog/digital conversion of the LCG signal, the total noise at the comparator input, $n_{LCG2}$, is given by

(7) $$\begin{array}{cc} n_{LCG2}^{2} & ={\left(\frac{C_{C}}{C_{C}+C_{ATN}}n_{pix}\left(t_{g}\right)\right)}^{2}-n_{LCG1}^{2}\\ \; & ={\left(\frac{C_{C}}{C_{C}+C_{ATN}}\right)}^{2}\left(n_{pix}^{2}\left(t_{e}\right)+n_{pix}^{2}\left(t_{g}\right)\right)+n_{cmp}^{2}\left(t_{e}\right) \end{array}$$

The noise $n_{LCG2}$ is lower than that of the baseline circuit (Figure 6) in which the voltage-follower noise is also imposed. The LCG noise is estimated using the sum of the AC noise simulation results with the pulse signal-setting at $t_{e}$ and $t_{g}$. The input-referred noise is given by $\frac{C_{C}+C_{ATN}}{C_{C}}n_{LCG2}$.

SPICE simulation results are summarized in Table 1. The input-referred HCG noise is given by $127.6\;\mu V_{\mathrm{rms}}\left(=\sqrt{\left(303.3^{2}+974.4^{2}\right)}/8\right)$. The input referred LCG noise is given by 545.9 $\mu V_{\mathrm{rms}}\left(=\sqrt{\left(39.9^{2}+391.0^{2}\right)}/0.72\right)$. Since the LCG noise of the baseline circuit is 652.6 $\mu V_{\mathrm{rms}}$, the LCG noise of the proposed double-sampling circuit is lower than that of the baseline circuit by 16.3%. The total capacitance and LCG noise of the baseline circuit and proposed double-sampling circuit is summarized in Table 2.

## 4. Fabrication and Evaluation of a Test Chip

**Figure 11:**
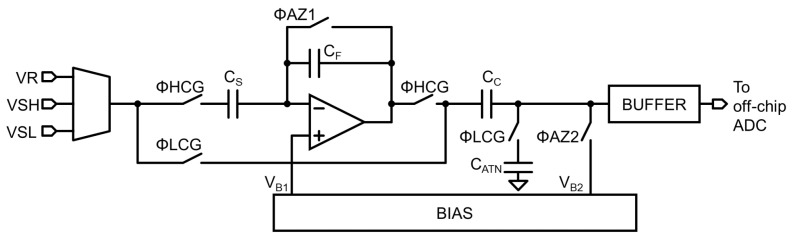
Test chip of the proposed up/down double-sampling circuit.

In order to verify the concept of the proposed read-out chain, a test circuit, which is shown in Figure 11, was fabricated with the 0.18μm 1P5M CMOS process and with MIM capacitors. The pseudo-pixel signal is supplied from external voltages, namely the HCG and LCG reset voltage VR, HCG signal voltage VSH, and LCG signal voltage VSL. An output buffer is implemented in the test chip to drive an off-chip 12-bit SAR-ADC.

**Figure 12:**
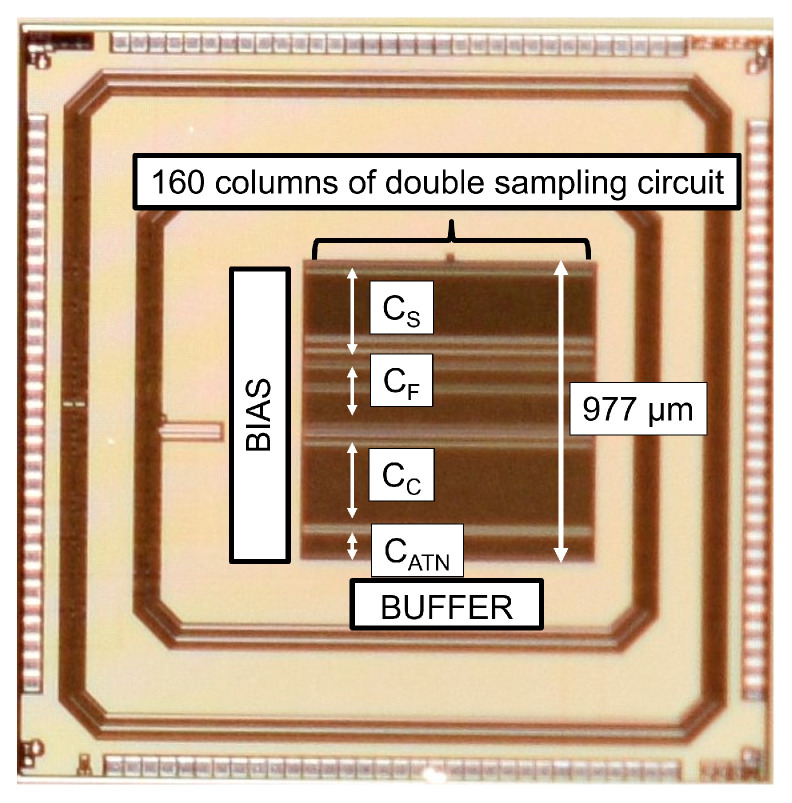
A photograph of the fabricated test chip.

A photograph of the fabricated test chip is shown in Figure 12. The 160 column of the up/down double-sampling circuits are laid in parallel to represent the layout constraint in the CMOS image sensor. The column pitch is 6.02 μm, which is obtained according to the minimum design rule of an MIM capacitor. The layout height of a column double sampling circuit is 977 μm. Since the capacitance density of the MIM capacitor is as low as 1.0 fF/μm${}^{2}$, 86% of the layout area of the column double-sampling circuit is occupied by the capacitors.

**Figure 13:**
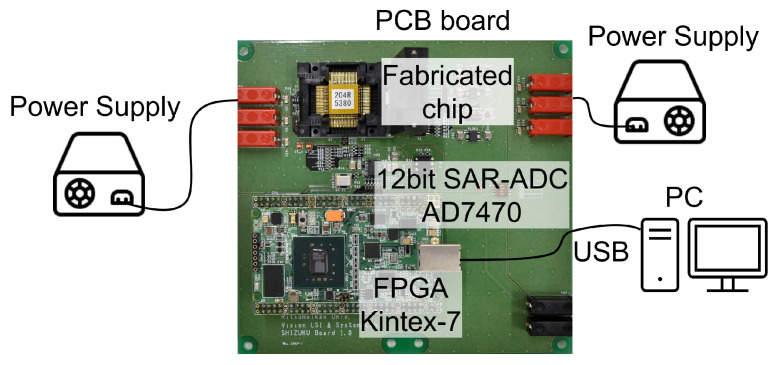
Measurement setup of the test chip.

The measurement setup of the test chip is shown in Figure 13. The fabricated chip, the off-chip 12-bit ADC, and an FPGA is mounted on a PCB board. The power supply voltage Vdd and pseudo-pixel voltage VR, VSH and VSL are supplied from external power sources. The control signal of the test chip is supplied by an FPGA. The digital output of the ADC was transferred to the PC via USB and then analyzed.

**Figure 14:**
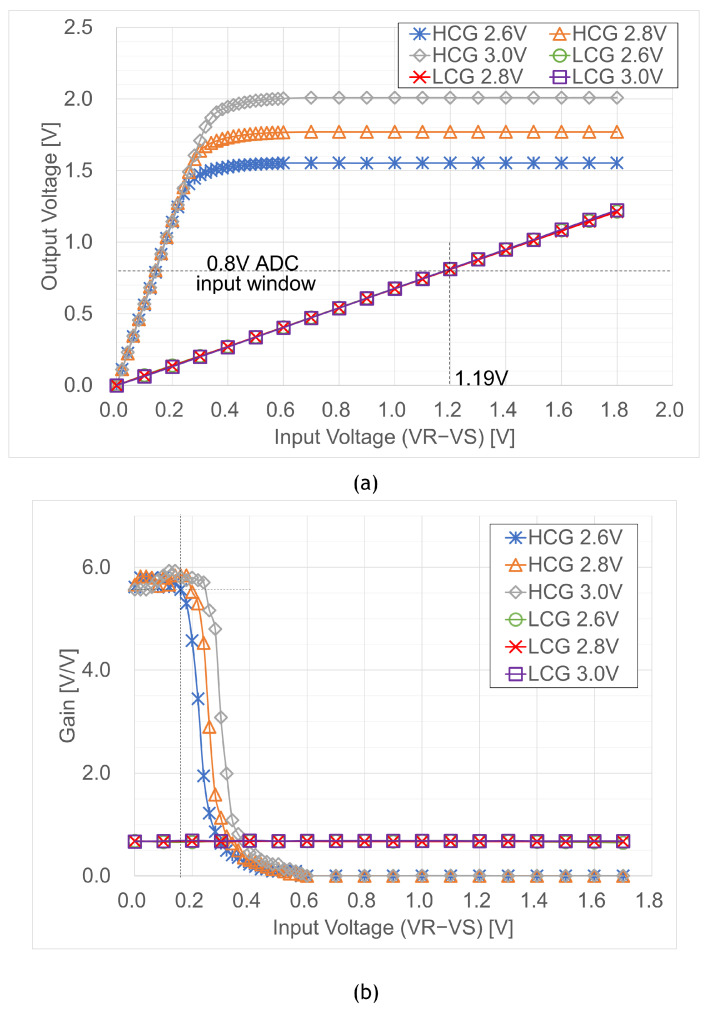
Measurement Results of input and output characteristics of the test chip. (**a**) Input and output characteristics of the up/down double-sampling circuit; (**b**) Gain of the double-sampling circuit.

Figure 14a shows the measured input and output characteristics, where the X-axis differs between reset and signal voltages, as it is VR−VSH for HCG or VR−VSL for LCG, and the Y-axis is the output voltage swing. The power supply voltage Vdd varied from 2.6 V to 3.0 V. The HCG output signal below a 0.8 V ADC input window shows high gains for a small input signal and is saturated for a large input signal. The LCG output signal is not saturated for a large input signal, and the input window of the double-sampling circuit is 1.19 V for a 0.8 V ADC input window. Figure 14b shows the gain in the double-sampling circuit derived from the input and output characteristics. The gain of the HCG signal was 5.58× for a small input signal, but lower than our target value of 8×. The root cause is supposed to be a parasitic capacitance caused in a narrow 6.02 μm pitch layout. The gain decreases as the output signal is saturated for a large input signal. The minimum confidence interval with a 90% slope is 0.18 V under a 2.6 V power supply voltage, and the minimum output range for the HCG signal is 1.03 V; these results are wider than a 0.15 V double-sampling input window and a 0.8 V ADC input window. The gain of the LCG signal is constant over a 1.19 V double-sampling input window. The measured gain was 0.67×, which is also lower than our target value of 0.72×.

Next, the measured noise for HCG and LCG is listed in Table 3. The input-referred dark noise values at 1σ are, respectively, 130.6 $\mu V_{\mathrm{rms}}$ at HCG and 452.9 $\mu V_{\mathrm{rms}}$ at LCG, which are close to the noise values from the SPICE simulation results, The HCG and LCG conversion gain of the LOFIC pixel are, respectively, supposed to be 160 $\mu {V/e}^{-}$, 10 $\mu {V/e}^{-}$ according to our previous work [16]. Considering the SF gain as 0.85×, the expected input-referred HCG noise at FD node is converted to $0.96\;e_{rms}^{-}$, while the 1.19 V LCG input window at FD node is converted to 140 ke${}^{-}$. Thus, the dynamic range of the proposed read-out chain is given by $20\log(140\times 10^{3}/0.96)=103\;\mathrm{dB}$. The specifications and performance of the proposed up/down double-sampling circuit are summarized in Table 4.

### Discussion

The test chip was fabricated with an MIM capacitor whose capacitance density is only 1.0 fF/μm${}^{2}$, and 86% of the layout area of the column double-sampling circuit was occupied by the capacitors. In a CMOS image sensor, a depletion MOS capacitor is typically utilized. For the case of a 3.3 V MOS transistor with a 75 Åoxide thickness, the capacitance density is 4.5 fF/μm${}^{2}$. Therefore, the layout height of the column double-sampling circuit will be reduced by 67% if the proposed double-sampling is implemented in LOFIC-CMOS image sensors.

The gain of the HCG signal was 5.58×, which is lower than our target value 8×. The root cause is supposed to be a parasitic capacitance. In the narrow 6.02 μm pitch layout, a wiring to the negative input of the amplifier and a wiring from the amplifier output are laid in parallel, and the parasitic capacitance between the wires increases the feedback capacitor $C_{F}$. According to Equation (2), the gain decreases as $C_{F}$ increases. The MIM capacitor of $C_{F}$ will be reduced considering the degradation of the gain in our future work. Furthermore, the higher-density MOS capacitor will decrease the length of the wire and will reduce the parasitic capacitor. The input-referred total noise will be reduced further by adjusting the gain to 8×. The gain of the LCG signal was 0.67× which is also lower than our target value 0.72×. As a result, the voltage swing at FD node is over the limit to transfer all PD charge to the FD node. The voltage swing at FD node will be decreased to the limit by adjusting the gain to 0.72×. Even though the LCG FWC on LCG will be decreased, the dynamic range will be kept, since the noise on HCG will also be decreased.

**Figure 15:**
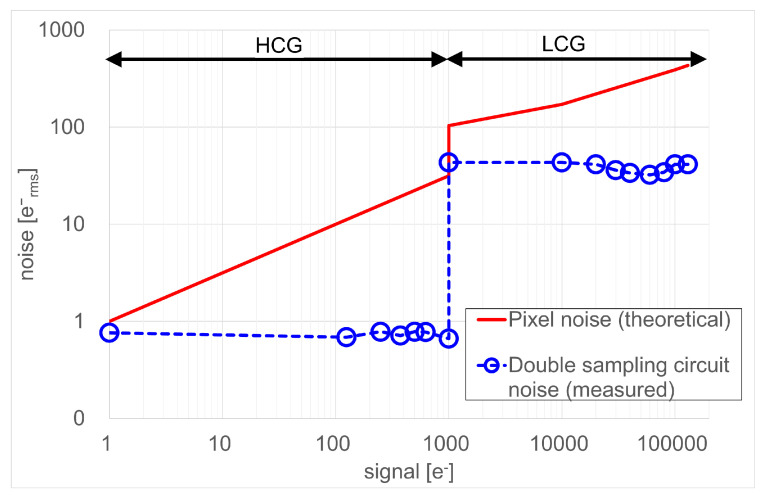
Measured double sampling circuit noise and theoretical pixel noise.

The noise of the double-sampling circuit is required to be less than the pixel noise. Figure 15 plots the measured double-sampling circuit noise and the theoretical pixel noise. In the HCG mode, the pixel noise plot is obtained by using the photon shot noise that follows $\sqrt{S}\;\left[e_{rms}^{-}\right]$ for the input signal $S\;[e^{-}]$. The measured double-sampling circuit noise is lower than the pixel noise. For input signals above 1.0 k e${}^{-}$, the HCG signal is saturated and the LCG signal is selected. In the LCG mode, the pixel noise is characterized as the photon shot noise and the FD reset noise, which is given by $\sqrt{S+kTC/q_{0}^{2}}\;\left[e_{rms}^{-}\right]$, where k is the Boltzmann constant, T is the absolute temperature, and C is the capacitance value of CFD and CS. The FD noise is not cancelled due to the non-correlated double-sampling operations. The double-sampling circuit noise is high in the LCG mode; however, this is still less than the pixel noise, which is $103\;e_{rms}^{-}$ at the transition point to the LCG mode.

## 5. Conclusions

An SEHDR-CMOS image sensor is realized with LOFIC pixels, in which the LCG signal and HCG signals are combined. However, the circuit area for the LOFIC pixel is large due to the two-channel read-out chain for polarity-inverted LCG and HCG signals. In order to realize a single-channel read-out chain and to reduce the chip cost of LOFIC-CMOS image sensors, an up/down double-sampling circuit composed of a inverting amplifier and non-inverting attenuator is proposed in this paper. Even though the polarity of the LCG and HCG signals output from the LOFIC pixel is inverted, the polarity of the input signal to a following ADC is the same and a single ADC can process both the HCG and LCG signals. Moreover, the attenuator is merged with the ADC to further decrease the area of the double-sampling circuit. The area of the proposed read-out chain is roughly reduced by 48% compared to the baseline two-channel read-out chain.

For LOFIC-CMOS image sensors, a voltage domain-level diagram is designed. The voltage gain of the double-sampling circuit in the HCG mode is selected to be 8× to decrease the input-referred circuit noise. The gain of the double-sampling circuit at the LCG mode is selected to be 0.72× to maximize FWC. In order to verify the concept of the proposed read-out chain, a test circuit is fabricated with a 0.18 μm CMOS process. According to the measurement results, the LCG input window of the double-sampling circuit is 1.19 V, while the input-referred dark noise for the HCG signal is 130.6 μV${}_{\mathrm{rms}}$. Supposing the pixel conversion gain of LOFIC-pixel, the dynamic range of the proposed read-out chain can be obtained at 103 dB using the expected input-referred HCG noise and LCG input window. It is also confirmed that the measured noise of the double-sampling circuit is less than the theoretical pixel noise throughout the input range of HCG and LCG.

## Figures and Tables

**Table 1:** AC noise simulation results.

**HCG**		
Noise @$t_{b}$	Noise @$t_{d}$	Input referred total noise
303.3 $\mu V_{\mathrm{rms}}$	974.4 $\mu V_{\mathrm{rms}}$	127.6 $\mu V_{\mathrm{rms}}$
**LCG**		
Noise @$t_{e}$	Noise @$t_{g}$	Input referred total noise
39.9 $\mu V_{\mathrm{rms}}$	391.0 $\mu V_{\mathrm{rms}}$	545.9 $\mu V_{\mathrm{rms}}$

**Table 2:** Comparison of the proposed read-out chain with the baseline read-out chain.

	Total Capacitance	Noise	
		HCG	LCG
Baseline	6.29 pF	122.1 $\mu V_{\mathrm{rms}}$	652.6 $\mu V_{\mathrm{rms}}$
Proposed	3.29 pF	127.6 $\mu V_{\mathrm{rms}}$	545.9 $\mu V_{\mathrm{rms}}$

**Table 3:** Measured double-sampling circuit characteristics, input-referred characteristics at FD node, and target pixel-conversion gain.

	Double-Sampling Circuit Characteristics		Input-Referred Characteristics at FD Node		Conversion Gain
	Noise	Input Window	Noise	Input window	
	[$\mu V_{\mathrm{rms}}$]	**[V]**	[$e_{\mathrm{rms}}^{-}$]	[$e^{-}$]	[$\mu V/e^{-}$]
HCG	130.6	0.15	0.96	1.0 k	160
LCG	452.9	1.19	53.3	140 k	10

**Table 4:** Performance summary of the test chip.

Process	0.18 μm 1P5M CMOS with MIM	
Supply voltage	2.6 V to 3.0 V	
Column pitch	6.02 μm	
# Columns	160	
	**HCG**	**LCG**
Measurement noise	130.6 μV${}_{\mathrm{rms}}$	452.9 μV${}_{\mathrm{rms}}$
Input window	0.15 V	1.19 V
Power consumption (sim)	23 μW @2.8 V	

## Data Availability

Not applicable.
